# The Effect of Miss and Tuck Stitches on a Weft Knit Strain Sensor

**DOI:** 10.3390/s21020358

**Published:** 2021-01-07

**Authors:** Emmanuel Ayodele, Syed Ali Raza Zaidi, Jane Scott, Zhiqiang Zhang, Maryam Hafeez, Des McLernon

**Affiliations:** 1 School of Electronic and Electrical Engineering, University of Leeds, Leeds LS2 9JT, UK; el15eoa@leeds.ac.uk (E.A.); Z.Zhang3@leeds.ac.uk (Z.Z.); D.C.McLernon@leeds.ac.uk (D.M.); 2 School of Architecture, Planning & Landscape, Newcastle University, Tyne NE1 7RU, UK; Jane.Scott@newcastle.ac.uk; 3 School of Computing and Engineering, University of Huddersfield, Huddersfield HD1 3DH, UK; M.Hafeez@hud.ac.uk

**Keywords:** weft knit sensor, miss stitches, tuck stitches, electromechanical modelling

## Abstract

Weft knitted conductive fabrics can act as excellent textile strain sensors for human motion capture. The loop architecture dictates the overall electrical properties of weft knit strain sensors. Therefore, research into loop architecture is relevant for comprehensively investigating the design space of e-textile sensors. There are three main types of knit stitches, Knitted loop stitch, Miss stitch, and Tuck stitch. Nevertheless, most of the research into weft knit strain sensors has largely focused on fabrics with only knitted loop stitches. Miss and tuck stitches will affect the contact points in the sensor and, consequently, its piezoresistivity. Therefore, this paper investigates the impact of incorporating miss and tuck stitches on the piezoresistivity of a weft knit sensor. Particularly, the electromechanical models of a miss stitch and a tuck stitch in a weft knit sensor are proposed. These models were used in order to develop loop configurations of sensors that consist of various percentages of miss or tuck stitches. Subsequently, the developed loop configurations were simulated while using LTspice and MATLAB software; and, verified experimentally through a tensile test. The experimental results closely agree with the simulated results. Furthermore, the results reveal that increases in the percentage of tuck or miss stitches in weft knit sensor decrease the initial and average resistance of the sensor. In addition, it was observed that, although the piezoresistivity of a sensor with tuck or miss stitches is best characterised as a quadratic polynomial, increases in the percentage of tuck stitches in the sensor increase the linearity of the sensor’s piezoresistivity.

## 1. Introduction

In the last decade, the application of knit fabrics has expanded from the traditional textile applications to their use in the creation of wearable electronics. From the use of warp knit to create textile antennas to the use of weft knit to create strain sensors, the application of knitting to create conventional electronics is being rapidly adopted [1,2,3,4,5,6]. In particular, conductive weft knitted fabrics have been utilised as strain sensors, because of their elastic structure and piezoresistivity [7,8,9,10,11]. The piezoresistivity of the sensor is the behaviour of the sensor’s electrical resistance when load is applied or the sensor is extended.

In recent past, several studies have investigated the impact of loop architecture on the piezoresistivity of the sensor. Notably, Atalay et al. intensively studied the impact of knitting parameters on a weft knit sensor’s piezoresistivity [12,13]. The sensors were created by knitting double covered elastomeric and silver-coated conductive nylon yarns in an interlock knit. Particularly, courses of the conductive nylon yarns were embedded on a host fabric. The host fabric was knitted in an interlock structure with elastomeric yarn. This sensor configuration was empirically selected by the authors for its high gauge factor and linearity. Subsequently, the effect of changes in (i) the input tension and linear density of the elastomeric yarn and (ii) the input tension of the conductive yarn were explored. It was observed that a decrease in the elastomeric yarn’s input tension or its linear density caused the electrical resistance to increase significantly. This was because they affect the number of contact points, which, in turn, affects the contact resistance. Moreover, the results showed that sensors that were knitted with a lower elastomeric yarn input tension exhibited a longer linear working range. Furthermore, the study illustrated that increases in the input tension of the conductive yarn caused an increase in the electrical resistance of the sensor. This occurred because an increase in the conductive yarn’s input tension reduced the stitch length. A reduced stitch length decreases the contact areas between the conductive loops due to the interlock structure of the host fabric. Consequently, the reduced contact areas increased the electrical resistance of the sensor. This phenomenon is consistent with Holm’s contact theory [14].

In a subsequent study, Atalay et al. [15] investigated the effect of the addition of elastomer. Two samples of sensors were manufactured in a plain knit. A sample of sensors was knitted with conductive yarn, while the other sample of sensors was knitted in a structure that comprised of elastomer and conductive yarn. It was observed that the sample knitted with only conductive yarn showed an inversely proportional relationship between its change in resistance and its extension, while the second sample with an elastic structure showed a directly proportional relationship between its change in resistance and its extension. This difference in piezoresistive behaviour occurred, because their electric circuits are fundamentally different. A major factor in the piezoresistive behaviour of a weft knit sensor is the contact resistance that occurs between two conductors. Particularly, in the sensor without the elastomer, contact resistance occurs at the intermeshing of one conductive loop with another conductive loop. Conversely, in the sensor with the elastomer, the interlocking of a conductive loop with a non-conductive loop does not create contact resistance. However, contact resistance still occurs in the sensor with the elastomer, because the elastomer increases the tightness of the fabric, such that the legs of the same conductive yarn loop make contact.

**Figure 1:**
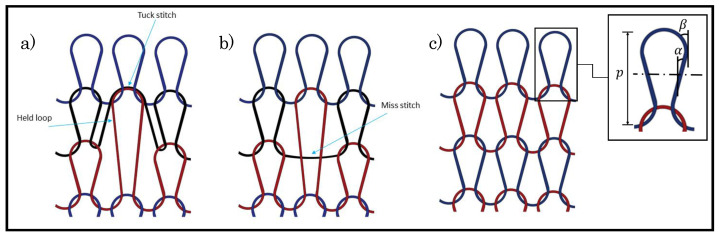
Types of loop stitches. (**a**) Tuck loop stitch, (**b**) Miss stitch, (**c**) Knitted loop stitch. The held loop is the knitted loop stitch tucked by a tuck stitch. *p* is the course spacing, α and β are the loop and interlocking angles, respectively.

In summary, changes in the knitting parameters have affected the piezoresistivity of a weft knit sensor. However, all of the previous studies mentioned have been implemented while only using a knitted loop stitch. In contrast, there are two other types of stitches, and they are miss stitch and tuck stitch. Figure 1 shows these three stitches. The tuck stitch occurs when a needle accrues more than one stitch thus tucking the extra stitch behind the first stitch. The extra stitch is the tuck stitch and it changes the structure of the fabric, because its legs are not connected to the head of a previous loop. A miss stitch materialises when a needle does not a collect a yarn, thus allowing for the yarn to float behind the needle and connecting the loops on either side of it [16]. These stitches can be combined with a knitted loop stitch to create different sensor configurations that may have different piezoresistive behaviour.

Therefore, in this study, we aim to investigate the effect of miss and tuck stitches on the piezoresistivity of a weft knit sensor. Particularly, we achieve this by proposing for the first time, detailed electro-mechanical models of miss and tuck stitches. These models are then simulated for loop configurations comprising of a varying percentage of miss stitches or tuck stitches in order to observe their impact on the sensor’s piezoresistive behaviour. In particular, we ensure that the effect of either miss or tuck stitches are separately observed by ensuring that the loop configurations only have miss stitches or tuck stitches. Thereafter, the predicted behaviour is experimentally validated by tensile testing sensors knitted with the same loop configurations.

## 2. Related Work

The effect of miss and tuck stitches on a weft knit sensor was described in the patent application [17]. Notably, the author describes a series of experiments, where the effect of miss and tuck stitches were studied on determining the optimal configuration for different applications. For these experiments, four samples of sensors were created by knitting conductive yarn in different percentages of miss and tuck stitches combined with a constant percentage of knitted loop stitches. A control sample was also knitted with only knitted loop stitches. The constant percentage of knitted loop stitches in each sample was 50%. The percentage ratio of miss stitches to tuck stitches (M2T) were (5:45), (10:40), (45:5), and (40:10).

The first experiment was implemented in order to determine the optimal structure for use in a resistive strain sensor. The parameters measured were the mean electrical resistance (MER), the dynamic range, i.e. maximum extension, and the effect of fabric thickness and optical porosity on the MER. The observations drawn from the experiment were:

1. The samples with M2T of (40:10) and (10:40) showed the largest dynamic range. This is excellent for strain applications, because the sensor can absorb the strain due to its flexibility.
2. The variation of resistance values was more stable in the four samples than in the control sample. This allows for more accurate measurements in strain sensing applications. The samples with the most stable resistance values were samples with M2T of (10:40) and (45:5).
3. Samples with a higher fabric thickness had a lower MER, while samples with a lower optical porosity had a lower MER. A lower MER is needed for an optimal regulation of the contact resistance, and this is achieved because a higher fabric thickness and lower optical porosity increase the contact area between the yarns. It was observed that all four samples had a lower MER when compared to the control sample with samples with M2T of (10:40) and (45:5) being the lowest.

Therefore, a strain sensor would be optimised if it is made with samples that show a large dynamic range and less variation in MER; and, they have a high fabric thickness and low optical porosity. The sample that fits this criterion is the sample with 10% miss stitches and 40% tuck stitches.

The second experiment involved placing two specific weights (150 gm and 400 gm) on the samples and then measuring the resistance of the sensor. This was then plotted with the baseline resistance before any weights were added. The plots show the linear fits of the resistances of the samples at the various weights (0, 150 and 400 gm) with varying negative gradients. The sensor with the highest coefficient of determination, $R^{2}$, was chosen as the optimal sample. The samples with the highest tuck stitches (M2T of (10:40) and (5:45)) were seen to have the highest $R^{2}$ value. It was assumed that this occurred because the tuck stitches increased the contact area and, thus, could regulate the contact resistance.

The third experiment involved human subjects of different weights placing their weights on the samples by standing with only one foot on the samples. The aim was to demonstrate the response of the sensor to the pressure from human concentrated weight in order to simulate what will happen in an application, such as socks that measure pressure of the feet. The experiment was only performed on samples with M2T of (5:45) and (10:40). The resistance was measured relative to the weight of each subject at different positions in the sensor and then plotted alongside the baseline resistance. The locations that were chosen to measure the resistance on the sensor were the points adjacent to the ankle and ball of the foot. The plot of the resistance relative to the weight provided a logarithmic response, unlike the linear response, which was obtained in the previous experiment. The optimal fabric was chosen on the basis of its gradient as a small gradient is favoured, because it illustrates a larger response to weight applied. The sensor with 10% miss stitches and 40% tuck stitches was found to have the smaller gradient and it was chosen as the optimal sensor.

Other experiments used a larger variation of M2Ts in the samples and, in one of them, samples were tested in order to deduce how resistance behaves relative to pressure that is applied in the wale and course directions. It was observed that, when pressure was applied in the course direction, there was no visible change in resistance. However, in the wale direction, there was a visible change in the resistance when pressure was exerted. The author also notes that the sensors with 10% miss stitches and 40% tuck stitches exhibit a solid inverse linear relationship between the resistance and load. This was attributed to the high percentage of tuck stitches as it increases the number of contact points. Another experiment sought to find out the relationship between the resistance of sensors with miss and tuck stitches and temperature changes. It was observed that the sensor displayed a linear relationship between its resistance and changes in its surrounding temperature. It was also observed that the samples with a higher number of tuck stitches had a better linear fit than other samples.

The experiments in this patent aim to ascertain whether the addition of miss and tuck stitches will optimise the resistance of the fabric for specific applications. This patent does an excellent job in illustrating how miss and tuck stitches can optimise a sensor for different applications. However, the conclusions are purely empirical and there is no theoretical model that describes the impact of the addition of miss and tuck stitches. Additionally, the rationale behind the choice of percentage of miss and tuck stitches in each loop configuration is not illustrated. Furthermore, combining miss and tuck stitches makes it more difficult to understand their separate impact on the piezoresistive behaviour of the sensor.

In contrast, the effect of miss and tuck stitches on a conductive weft knit fabric were investigated separately [18]. The samples were knitted with knit stitches and different percentages of either miss or tuck stitches. Subsequently, their resistance was measured and it was observed that the increases in the percentage of tuck or miss stitches caused a decrease in the resistance of the sensor. However, this study is only experimental as there is no theoretical model to explain the cause of the impact. In addition, this study was limited to the impact of miss and tuck stitches on the initial resistance of the sensor. It did not investigate the impact of miss and tuck stitches on the sensor’s piezoresistivity.

Furthermore, Liu et al. [19] also investigated the impact of consecutive miss stitches on the overall resistance of a conductive weft knit fabric. Samples of the fabric were knitted in a plain base structure with a course containing one knitted loop and varying numbers of consecutive miss stitches. It was observed that, as the number of consecutive miss stitches increased, the resistance of the fabric decreased. This effect materialised because of the reduced contact resistance that is caused by the miss stitches. However, this study only investigates the impact on the initial resistance of the sensor and not its behaviour when the sensor is extended.

This paper addresses the research gap that is neglected in previous studies. Notably, we investigate the effect of miss and tuck stitches on a weft knit sensor separately. Furthermore, unlike previous studies, we do not limit our study to only the initial resistance of the sensor. We also investigate the piezoresistivity of the sensors as they are extended. Particularly, the mean resistance, as well as the linear and quadratic $R^{2}$ values of the sensor’s piezoresistivity. In addition, we do not restrict this study to only experimental observations. In contrast, we propose electromechanical models that explain the behaviour of the sensors.

## 3. Materials and Methods

### 3.1. Electromechanical Model of a Tuck Stitch

This section describes a novel resistive model of a tuck stitch in a weft knit sensor. The basic assumptions used to formulate this model are:

- The conductive yarn used is a perfect intrinsic conductor.
- The lengths of the head of a tuck stitch and the head of its held knitted loop stitch are equal.
- The head and sinker of a knitted loop stitch are of equal lengths.

**Figure 2:**
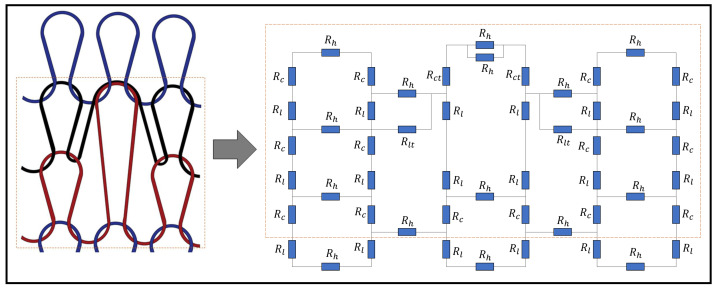
The resistive model of a Tuck stitch in a Weft Knit Sensor.

Figure 2 illustrates the theoretical model of a tuck stitch that is knitted with conductive yarn. In this model, we postulate that a tuck stitch adds length resistances as a result of its legs and heads. Furthermore, we claim that it changes the contact resistance between the loops, because of the contact pressure that it adds to the fabric, especially at the location of the tuck stitches. Particularly, plain knit fabrics with tuck stitches are known to be less extensible than plain knit fabrics without tuck stitches, because the tucked loops add an extra layer of pressure at the junctions where the intermeshing of loops occurs [16]. Therefore, by representing its geometrical parameters with equivalent resistive values, we model the tuck stitch as a resistive circuit.

While using Postle model [20], we consider the loop leg, $L_{l}$, as a bent beam and derive its length as:

(1) $$L_{l}=\frac{p}{\sqrt{2(\sin\alpha +\sin\beta )}}f\left(k,\gamma \right),$$

where f(k,γ) is the difference between the complete and incomplete integrals, and it can be calculated as

(2) $$f\left(k,\gamma \right)=\int_{0}^{\frac{\pi }{2}}\frac{d\gamma }{\sqrt{1-k^{2}\sin^{2}\gamma }}-\int_{0}^{\gamma }\frac{d\gamma }{\sqrt{1-k^{2}\sin^{2}\gamma }},$$

and parameters *k* and γ are calculated as:

(3) $$k=\sin\left(\frac{\pi }{4}+\frac{\alpha }{2}\right),$$

(4) $$\gamma =\sin^{-1}\left(\frac{1}{k\sqrt{2}}(\cos\frac{\beta }{2}-\sin\frac{\beta }{2}\right)),$$

The length of the loop head, $L_{h}$, is also calculated while using the Postle model. By considering it to be two equal segments of a circle, we derive it as:

(5) $$L_{h}=\frac{p(\frac{\pi }{2}-\beta )}{2(\sin\alpha +\sin\beta )},$$

The resistance of the held loop’s legs and header is then calculated as:

(6) $$R_{l}=\frac{\rho L_{l}}{A_{r}},$$

(7) $$R_{h}=\frac{\rho L_{h}}{A_{r}},$$

where $A_{r}$ is the cross-sectional area of the conductive yarn.

As mentioned in the earlier assumption, we model the length of tuck stitch head to be equal to the length of the held loop head. Therefore, the resistances of tuck and held loop heads are the same. Consequently, a parallel connection of resistors is formed. The combination of this resistances at the head of the tuck stitch is calculated as:

(8) $$R_{ht}=\frac{R_{h}}{2}.$$

Using the Kurbak model [21], the total length of a tuck stitch, $L_{tt}$ can be calculated:

(9) $$L_{tt}=L_{t}-4d,$$

where *d* is the diameter of the yarn. Furthermore, from the Munden model [22], the length of a stitch is:

(10) $$L_{tt}=2\left(L_{ht}+L_{lt}\right),$$

Therefore because the loop length of the held loop head is equal to the tuck loop head, the length of the tuck loop leg is

(11) $$L_{lt}=L_{l}-2d,$$

and its resistance is

(12) $$R_{lt}=\frac{\rho L_{lt}}{Ar}.$$

The contact resistance in the tucked loop is determined by assuming that the contact pressure is twice the contact pressure at a knit loop, because both the tuck yarn and held yarn interlock the previous loop. Therefore, by combining this assumption with Holm’s contact theory, the contact resistance at a tuck yarn, $R_{ct}$, is derived as:

(13) $$R_{ct}=\frac{\rho }{2}\sqrt{\frac{\pi H}{2nP_{r}}},$$

where $P_{r}$ is the contact pressure between the loops, *n* is the number of contact points, *H* is the material hardness, and ρ is the resistivity.

Therefore, the contact resistance at a tuck loop can be related to the contact resistance at a knit loop as:

(14) $$R_{ct}=0.707*R_{c}.$$

### 3.2. Electromechanical Model of a Miss Stitch

**Figure 3:**
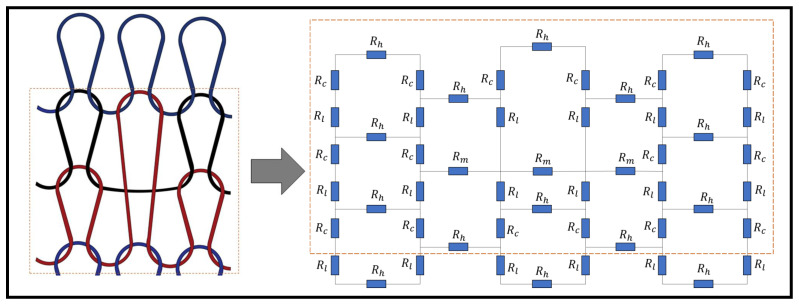
Resistive model of a Miss stitch in a Weft Knit Sensor.

Figure 3 illustrates a novel resistive model of a weft knit sensor with a miss stitches. In order to model this sensor, it was assumed that the miss stitch is split across three equal lengths as it floats from one interlocked loop to another.

In modelling a miss stitch, the contact resistance present in a knitted loop is removed because there are no interlocking loops. However, the resistance of a miss stitch can be modelled as a length resistance. Therefore, we propose that the length of a miss stitch is the sum of all wale spacings of all the loops that it floats across. Therefore, for a miss stitch that floats across one loop, its length, $L_{m}$, can be described as:

(15) $$L_{m}=W_{s}.$$

where $W_{s}$ is the average wale spacing of the fabric.

Furthermore, based on the assumption that length of a miss stitch is split equally in three lengths as it extends from one interlocked loop to another, we introduce a parameter $R_{m}$ in order to represent the resistance of one-third of the miss stitch. Therefore, the resistance parameter, $R_{m}$, was calculated as:

(16) $$R_{m}=\frac{\rho W_{s}}{3Ar}.$$

### 3.3. Circuit Analysis

**Figure 4:**
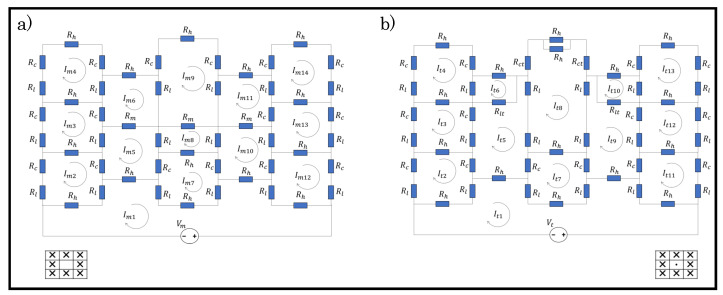
Resistive circuits of (**a**) a Miss stitch, and (**b**) a tuck stitch in a Weft Knit Sensor.

A circuit analysis is undertaken in order to determine the equivalent resistance of a weft knit sensor with tuck stitches or miss stitches. To achieve this, two sensors with three wales and three courses are illustrated in Figure 4, where a miss stitch and a tuck stitch are located in the middle of the sensors. The kirchoff current and voltage laws were employed in order to derive the equivalent resistance in the sensors.

In order to derive the equivalent resistance in the sensor with the miss stitch, we add a voltage source and use the hypothetical currents ($I_{m1}-I_{m14}$) to determine the equivalent resistance.

(17) $$\begin{array}{c} \left(I_{m1}-I_{m2}\right)\left(R_{h}+R_{l}\right)+\left(I_{m1}-I_{m5}\right)R_{h}+\left(I_{m1}-I_{m7}\right)\left(2R_{l}+R_{h}\right)+\\ \left(I_{m1}-I_{m10}\right)R_{h}+\left(I_{m1}-I_{m12}\right)\left(R_{h}+R_{l}\right)=0, \end{array}$$

(18) $$\begin{array}{c} \left(I_{m2}-I_{m1}\right)\left(R_{h}+R_{l}\right)+I_{m2}\left(R_{c}+R_{l}\right)+\left(I_{m2}-I_{m3}\right)R_{h}+\left(I_{m2}-I_{m5}\right)R_{c}=0, \end{array}$$

(19) $$\begin{array}{c} \left(I_{m3}-I_{m5}\right)R_{l}+\left(I_{m3}-I_{m2}\right)R_{h}+I_{m3}\left(R_{c}+R_{l}\right)+\left(I_{m3}-I_{m4}\right)R_{h}+\left(I_{m3}-I_{m6}\right)R_{c}=0, \end{array}$$

(20) $$\begin{array}{c} \left(I_{m4}-I_{m6}\right)R_{l}+\left(I_{m4}-I_{m3}\right)R_{h}+I_{m4}\left(2R_{c}+R_{l}+R_{h}\right)=0, \end{array}$$

(21) $$\begin{array}{c} \left(I_{m5}-I_{m6}\right)R_{m}+\left(I_{m5}-I_{m8}\right)R_{l}+\left(I_{m5}-I_{m7}\right)R_{c}+\left(I_{m5}-I_{m1}\right)R_{h}+\\ \left(I_{m5}-I_{m2}\right)R_{c}+\left(I_{m5}-I_{m3}\right)R_{l}=0, \end{array}$$

(22) $$\begin{array}{c} I_{m6}\left(R_{h}\right)+\left(I_{m6}-I_{m9}\right)R_{l}+\left(I_{m6}-I_{m5}\right)R_{m}+\left(I_{m6}-I_{m3}\right)R_{c}+\left(I_{m6}-I_{m4}\right)R_{l}=0, \end{array}$$

(23) $$\begin{array}{c} \left(I_{m7}-I_{m1}\right)\left(R_{h}+2R_{l}\right)+\left(I_{m7}-I_{m5}\right)R_{c}+\left(I_{m7}-I_{m8}\right)R_{h}+\left(I_{m7}-I_{m10}\right)R_{c}=0, \end{array}$$

(24) $$\begin{array}{c} \left(I_{m8}-I_{m7}\right)R_{h}+\left(I_{m8}-I_{m5}\right)R_{l}+\left(I_{m8}-I_{m9}\right)R_{m}+\left(I_{m8}-I_{m10}\right)R_{l}=0, \end{array}$$

(25) $$\begin{array}{c} \left(I_{m9}-I_{m11}\right)R_{l}+\left(I_{m9}-I_{m8}\right)R_{m}+\left(I_{m9}-I_{m6}\right)R_{l}+I_{m9}\left(R_{h}+2R_{c}\right)=0, \end{array}$$

(26) $$\begin{array}{c} \left(I_{m10}-I_{m13}\right)R_{l}+\left(I_{m10}-I_{m12}\right)R_{c}+\left(I_{m10}-I_{m1}\right)R_{h}+\\ \left(I_{10}-I_{m7}\right)R_{c}+\left(I_{m10}-I_{m8}\right)R_{l}+\left(I_{m10}-I_{m11}\right)R_{m}=0, \end{array}$$

(27) $$\begin{array}{c} I_{m11}\left(R_{h}\right)+\left(I_{m11}-I_{m14}\right)R_{l}+\left(I_{m11}-I_{m13}\right)R_{c}+\\ \left(I_{m11}-I_{m10}\right)R_{m}+\left(I_{m11}-I_{m9}\right)R_{l}=0, \end{array}$$

(28) $$\begin{array}{c} \left(I_{m12}-I_{m1}\right)\left(R_{h}+R_{l}\right)+\left(I_{m12}-I_{m10}\right)R_{c}+\left(I_{m12}-I_{m13}\right)R_{h}+I_{m12}\left(R_{c}+R_{l}\right)=0, \end{array}$$

(29) $$\begin{array}{c} \left(I_{m13}-I_{m10}\right)R_{l}+\left(I_{m13}-I_{m11}\right)R_{c}+\left(I_{m13}-I_{m14}\right)R_{h}+\\ I_{m13}\left(R_{c}+R_{l}\right)+\left(I_{m13}-I_{m12}\right)R_{h}=0, \end{array}$$

(30) $$\begin{array}{c} \left(I_{m14}-I_{m11}\right)R_{l}+I_{m4}\left(2R_{c}+R_{l}+R_{h}\right)+\left(I_{m14}-I_{m13}\right)R_{h}=0. \end{array}$$

The hypothetical currents were calculated as

(31) $$i=R^{-1}v,$$

where,

$$i=\left[\begin{array}{c} I_{m1}\\ I_{m2}\\ \ldots \\ I_{m14} \end{array}\right],v=\left[\begin{array}{c} V_{m}\\ 0\\ \ldots \\ 0 \end{array}\right]$$

and

$$R=\left[\begin{array}{cccccc} 5R_{h}+4R_{l} & \ldots & 0 & 0 & \ldots & 0\\ -(R_{h}+R_{l}) & \ldots & 0 & 0 & \ldots & 0\\ \ldots & \ldots & \ldots & \ldots & \ldots & \ldots \\ 0 & \ldots & 0 & -R_{l} & \ldots & 0\\ -2R_{l}+R_{h} & \ldots & -R_{h} & 0 & \ldots & 0\\ 0 & \ldots & R_{m}+R_{h}+2R_{l} & -R_{m} & \ldots & 0\\ 0 & \ldots & -R_{m} & 2R_{l}+2R_{c}+R_{m}+R_{h} & \ldots & 0\\ \ldots & \ldots & \ldots & \ldots & \ldots & \ldots \\ 0 & \ldots & 0 & 0 & \ldots & 2(R_{c}+R_{h}+R_{l}) \end{array}\right].$$

The equivalent resistance was then calculated as:

(32) $$R_{m(eq)}=\frac{V_{m}}{I_{m1}}.$$

In order to derive the equivalent resistance in the sensor with the tuck stitch, we employ the same methodology in determining the equivalent resistance. The hypothetical currents $I_{tn}$ were derived while using (31), but resulted in a different resistance matrix. The resistance matrix derived for the sensor with a tuck stitch was calculated as:

$$R=\left[\begin{array}{ccccccc} 5R_{h}+4R_{l} & \ldots & -R_{h} & \ldots & 0 & \ldots & 0\\ -(R_{h}+R_{l}) & \ldots & -R_{c} & \ldots & 0 & \ldots & 0\\ \ldots & \ldots & \ldots & \ldots & \ldots & \ldots & \ldots \\ -R_{h} & \ldots & 3R_{l}+3R_{c}+R_{h}+R_{lt} & \ldots & -2R_{l} & \ldots & 0\\ 0 & \ldots & -R_{lt} & \ldots & 0 & \ldots & 0\\ -(2R_{l}+R_{h}) & \ldots & -R_{c} & \ldots & -R_{h} & \ldots & 0\\ 0 & \ldots & -2R_{l} & \ldots & 4R_{l}+2R_{ct}+1.5R_{h} & \ldots & 0\\ \ldots & \ldots & \ldots & \ldots & \ldots & \ldots & \ldots \\ 0 & \ldots & 0 & \ldots & 0 & \ldots & 2(R_{c}+R_{h}+R_{l}) \end{array}\right],$$

and the equivalent resistance was calculated as:

(33) $$R_{t(eq)}=\frac{V_{t}}{I_{t1}}.$$

### 3.4. Determination of Contact Resistance

The conductive yarn used in the simulation and fabrication of the sensor is a multifilament yarn consisting of 20% stainless steel and 80% polyester filaments. Consequently, the stainless steel component ensures that the sensor complies to Holm’s contact theory which postulates that the contact resistance between two conductors can be calculated as:

(34) $$R_{c}=\frac{\rho }{2}\sqrt{\frac{\pi H}{nP_{r}}},$$

where, $R_{c}$ is the contact resistance, ρ is the electrical resistivity, *H* is the hardness of the material used, *n* is the number of contact points, and $P_{r}$ is the contact pressure between the conducting materials.

The electrical resistivity and the material hardness are constant properties of the conductive yarn, while the sensor’s design determines the number contact points. Therefore, the contact resistance is mainly dependent on the contact pressure between the loops. In particular, changes in the contact pressure across the loops during the extension of the sensor causes changes in the contact resistance and consequently the piezoresistivity of the sensor. However, the contact pressure between the interlocking loops has proven to be difficult to measure or predict. Therefore, researchers have found alternative methods to accurately determine the contact resistance by measuring the contacting force [4].

However, Zhang et al. [9] proposed, from empirical observations, that the correlation between the contact resistance, $R_{c}$, and equivalent resistance, $R_{eq}$, of the sensor can be derived as:

(35) $$R_{eq}=R_{c}\cdot K,$$

where *K* is a variable coefficient that is based on the sensor’s structural design.

From Equations (31) and (32), we derive that

(36) $$R_{1,1}^{-1}=R_{eq},$$

where $R_{1,1}^{-1}$ is the first element of the inverse matrix of the resistance matrix R of the sensor. Therefore,

(37) $$R_{1,1}^{-1}=D\cdot R_{c}.$$

where *D* is the coefficient of $R_{c}$ in $R_{1,1}^{-1}$.

However, this method is computationally intensive. Therefore, we propose a less computationally intensive method in Algorithm 1. Algorithm 1 is a control algorithm that calculates the contact resistance from the equivalent resistance with an initial random positive value as *D*. Subsequently, a control feedback is used in order to calculate the optimised value of *D*. A threshold was set to stop the algorithm when the percentage change in the input contact resistance to its previous value was less than 3%. This was selected empirically as the accuracy of the model was not significantly improved below this threshold.
**Algorithm 1** Contact Resistance Solution***Initialise***:$R_{sim}\leftarrow 0$D←0<D<inf***Loop***:$R_{c}=R_{exp}/D$Input $R_{c}$ into modelled circuit to determine $R_{sim}$**if**$|R_{sim(n)}-R_{sim(n-1)}|>\left(0.03\cdot R_{sim(n)}\right)$**then**    $D=R_{sim}/R_{c}$    **goto** ***Loop*****else**Return $R_{c}$**end if**                      ▹$R_{sim}$ and $R_{exp}$ are the simulated and experimental equivalent resistances respectively

### 3.5. Simulation Parameters

**Figure 5:**
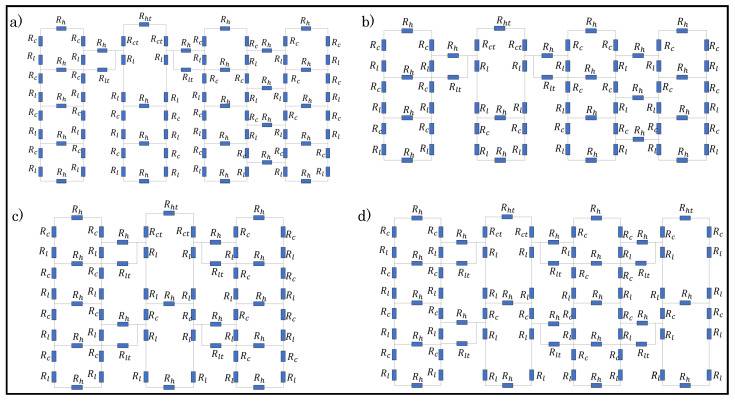
Unit circuit diagram of samples with tuck stitches. (**a**) 6.25% tuck stitches (**b**) 8.33% tuck stitches (**c**) 16.67% tuck stitches (**d**) 25% tuck stitches.

**Figure 6:**
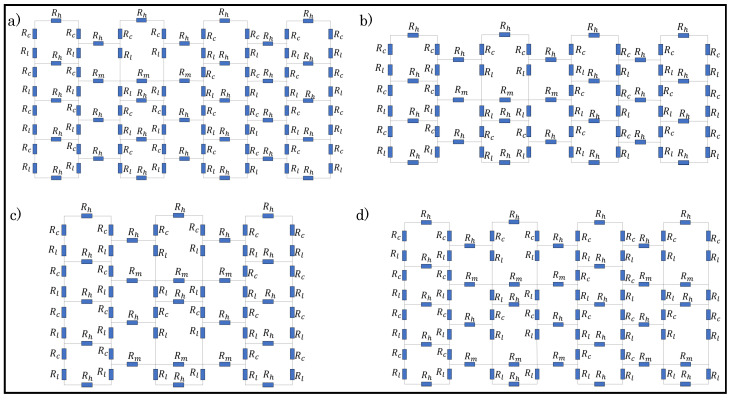
Unit circuit diagram of samples with miss stitches. (**a**) 6.25% miss stitches; (**b**) 8.33% miss stitches; (**c**) 16.67% miss stitches (**d**) 25% miss stitches.

Dfferent loop configurations of a sensor with varying percentages of tuck stitches and miss stitches within the sensor were designed in order to determine the effect of miss and tuck stitches on a plain weft knit sensor. The loop configurations depicted in Table 1 were then simulated whlie using the postulated models. Figure 5 and Figure 6 show the unit circuit diagram of the loop configurations. It can be observed in the circuit diagram of sensors with 6.25% and 8.33% tuck stitches that the length resistances of the sinker loops of the held loop were neglected. It was initially assumed that their lengths were equal to the lengths of the sinker loop of other knit stitches. However, it was observed that this assumption negatively affected the expected results. In contrast, neglecting it significantly improved the simulation results of the model in terms of its correlation with experimental results. A feasible explanation for this occurrence is that the lengths of the sinker loops of the held loop are negligible, because they have transferred to the lengths of the loop legs of the held loop, thus resulting in the longer loop legs of a held loop than other knitted loop stitches.

Moreover, the loop configurations were designed in order to prevent consecutive tuck or miss stitches either in the course or wale direction to reduce the complexity in modelling. These loop configurations were simulated with LTspice using the numerical variables that are shown in Table 2.

### 3.6. Experimental Validation

#### 3.6.1. Sample Preparation

Eight samples of weft knit sensors were knitted while using a Shima Seiki Mach2s 12-gauge knitting machine with the loop configurations shown in Table 1. All of the sensors were knitted with conductive yarn as a 72 courses by 72 wales plain knit fabric with a digital cam setting of 30. The cam setting is a dimensionless value that represents the stitch length that will be attempted by the knitting machine. The conductive yarn used in knitting the samples was a Schoeller multifilament conductive yarn that is commercially available from Uppingham Yarns Ltd. According to its specification sheet, it can be stretched up to 5.5% extension and its yarn count and linear density were 2/50 Nm and 400 dtex, respectively. Notably, our simulation parameter for resistivity was chosen as 300 Ωmm after a preliminary measurement of a relaxed sample of the yarn, as seen in Table 2. Furthermore, the yarn consists of 80% polyester and 20% stainless steel filaments that provide an advantage of being an intrinsic conductor as opposed to yarns that are coated with conductive ink. Particularly, a multifilament conductive yarn is more environmentally stable than a coated conductive yarn, because the conductive inks used in coating yarns are very sensitive to environmental changes, such as temperature [23].

**Figure 7:**
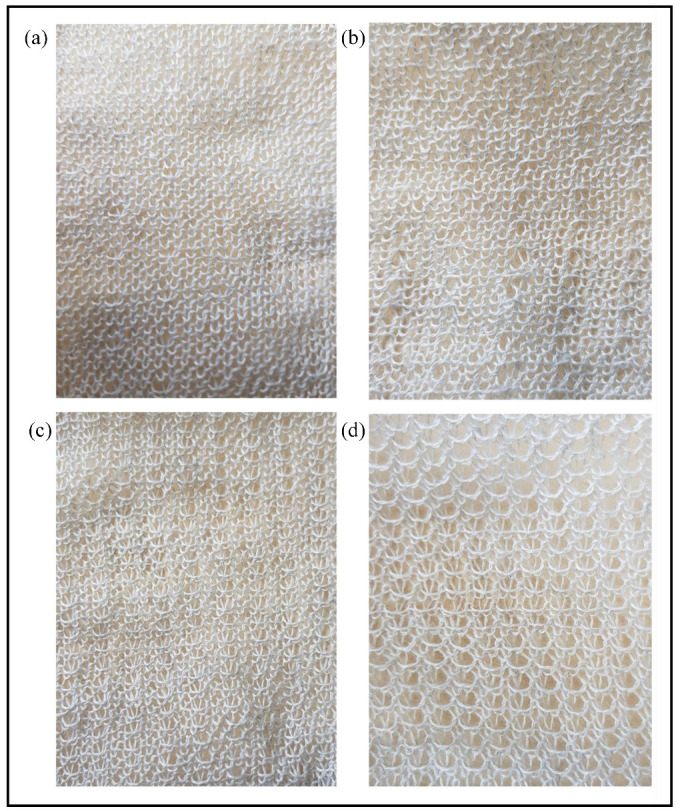
Knitted samples with miss stitches. (**a**) 6.25% miss stitches (**b**) 8.33% miss stitches (**c**) 16.67% miss stitches (**d**) 25% miss stitches.

**Figure 8:**
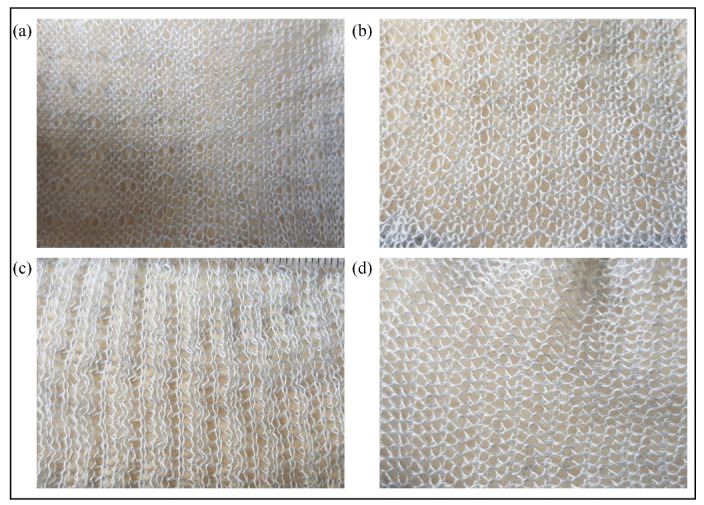
Knitted samples with tuck stitches. (**a**) 6.25% tuck stitches (**b**) 8.33% tuck stitches (**c**) 16.67% tuck stitches (**d**) 25% tuck stitches.

Figure 7 and Figure 8 illustrate the knitted samples and Table 3 shows their respective parameters. Tuck stitches reduce the length of a fabric and increase the width of a fabric. This is illustrated in Table 3, where the reduced length and increased width caused a larger number of courses/cm and a smaller number of wales/cm as the percentage of tuck stitches in the sensor increased. The reduced length and increased width occur, because the tension of tuck stitches pull down their held loops, thereby decreasing their length, but expanding their width [16]. In contrast, miss stitches reduce the width of the fabric, because the wales are more drawn together by the miss stitches. This phenomenon is observed in all sensor configurations with miss stitches except the sensor comprising of 6.25% miss stitches, as depicted in the changes in the wales/cm. The exception is likely a result of the location of the miss stitches. In addition, miss stitches also reduce the length of the fabric, as illustrated by the increase in courses/cm of the sensors as the percentage of miss stitch increased. This occurred, because, in a miss stitch, the loop height is replaced by the diameter of the yarn, which, in most cases, is considerably smaller than the loop height.

#### 3.6.2. Experimental Procedure

The samples were dry relaxed for 48 h in order to remove any existing strains from the knitting process. Thereafter, a tensile test was performed while using an Instron3369 tensile machine. Particularly, the steel clamps were lined with insulated rubber pads in order to prevent any conductance between the tensile machine and the sensors. The testing procedure consisted of stretching the sensors in the course-wise direction at a speed of 10 mm per minute until the sensors were extended to 25% extension, while the sensor’s resistance was measured by a digital multimeter (TENMA 72-7770a).

## 4. Results and Discussion

### 4.1. Effect of Tuck Stitches on a Weft Knit Sensor

**Figure 9:**
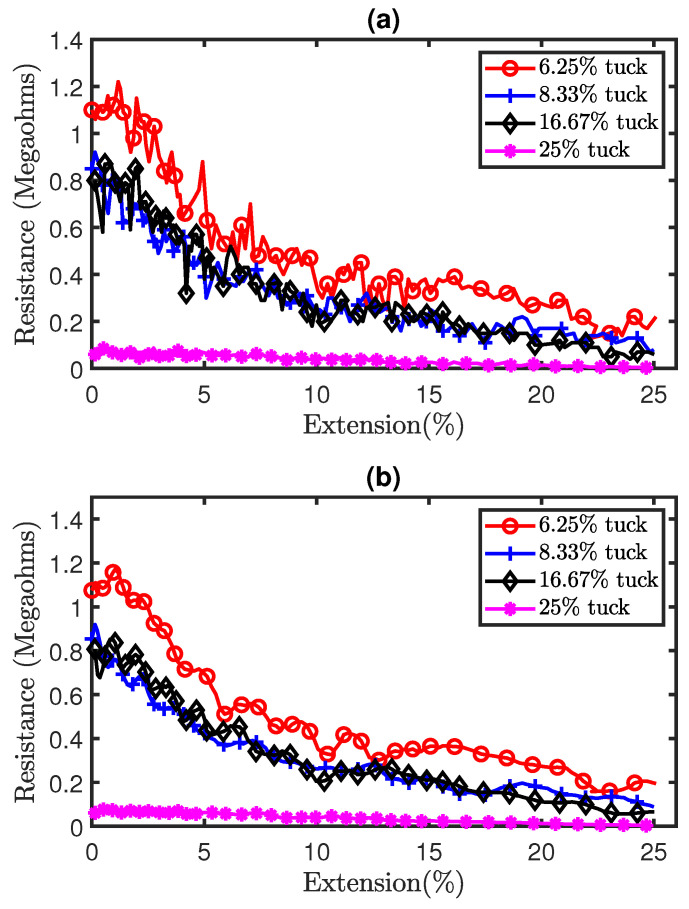
Experimental results of tensile test on sensors with tuck stitches. (**a**) Pre-filtered results, (**b**) Post-filtered results.

**Figure 10:**
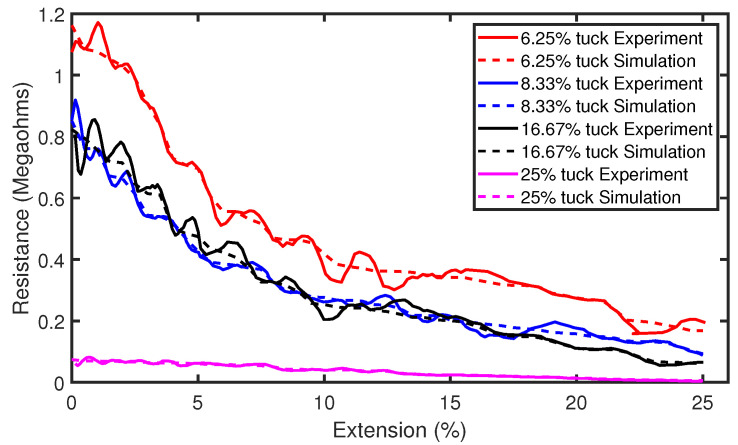
Comparison of simulation and experimental results for sensors with tuck stitches.

Part (a) of Figure 9 shows the raw experimental results of the tensile experiment with sensors consisting of tuck stitches. It can be observed that there is a significant level of analog noise in the data seen in the variations of resistance as the sensors were extended. This was caused by the hysteresis that is present in weft knit strain sensors [12]. Therefore, we applied a Savitzky–Golay filter of polynomial order N = 5 and window length of 9 to reduce the analog noise from the data. The filtered data are illustrated in part (b) of Figure 9. Furthermore, the results show a decrease in the resistance as the sensors are stretched. This occurred, because, when a weft knit strain sensor is stretched, the contact pressure between the loops increases, causing the contact resistance to decrease, as observed in Holm’s contact theory [14]. In addition, Figure 10 shows a comparison of the experimental and simulation results. It can be observed that our model accurately simulates the piezoresistive behaviour of the sensors.

**Figure 11:**
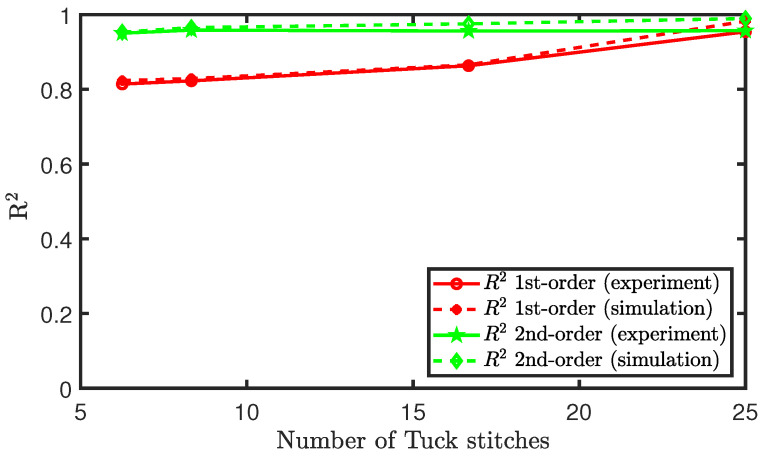
Polynomial fit of the piezoresistive behaviour of sensors with tuck stitches.

Observing these results, it is difficult to ascertain whether the sensor piezoresistive behaviour can be characterised as a polynomial of the first-order (linear) or a polynomial of the second-order (quadratic). Therefore, we plot the $R^{2}$ value, coefficient of determination, of both polynomial fits for each configuration of tuck stitches in the sensor. The $R^{2}$ values of both polynomial fits were both higher than 0.8, but the results show that the quadratic polynomial is a better fit, as shown in Figure 11. However, we observe that, as the percentage of tuck stitches in the sensor increases, the $R^{2}$ value of the first-order polynomial fit increases. In simple terms, this means that the increase in tuck stitches led to a more linear piezoresistive behaviour in the sensor. Particularly, the sensor with 25% tuck stitches exhibited a higher $R^{2}$ value in its linear fit than the quadratic fit. Moreover, the $R^{2}$ value of the linear fit of the sensors was observed to be 0.954 for the sensor with 25% tuck stitches. This is much higher than comparable weft knit strain sensors that were knitted without the miss or tuck stitches reported in Atalay et al. [15] where $R^{2}$ values of 0.6652–0.816 were observed. This effect occurred, because, as illustrated in Equation (14), the addition of tuck stitches increases the contact pressure at the contact points. Increased pressure at the contact point has been observed to cause a higher linear piezoresistive behaviour in weft knit sensors [12]. This phenomenon occurs because the larger initial contact pressure increases the extension range for the intermesh between the loops to disintegrate during extension, thereby preserving the linear decrease of the contact area as the sensor is extended. Furthermore, it can be observed that our simulation results closely agree with the experimental results.

**Figure 12:**
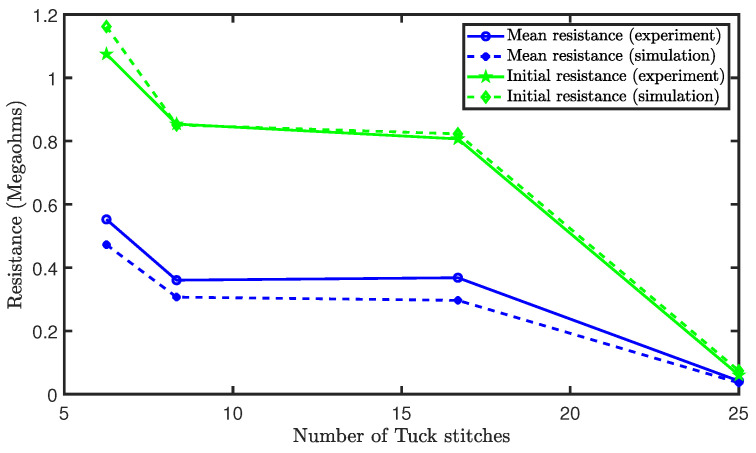
Initial and mean resistances of sensors with tuck stitches.

Figure 12 shows the initial and mean resistances. It was observed that increases in the tuck stitches led to a decrease in the initial resistance of the sensor. This occurred because an increase in the percentage of tuck stitches causes an increased initial contact pressure. This increase in contact pressure reduces the contact and equivalent resistances, as mentioned earlier. The mean resistance across the extension is also depicted in Figure 12. There was a reduction in the mean resistance as the percentage of tuck stitches in the sensor increases with the exception of the sensor with 16.67% tuck stitches where the mean resistance slightly increased. It is believed that this exception is a result of the limitations of the loop configuration and that a different loop configurations with the same percentage of tuck stitches will have a lower mean resistance during extension than sensors with a lower percentage of tuck stitches. In particular, the sensor with 16.67% tuck stitches is the only configuration (with tuck stitches) with a courses/wales ratio (1.33) > 1. Increases in courses/wales ratio increases the resistance of the sensor, as observed by Li et al. [24]. Therefore, we believe that the higher resistance in sensor with 16.67% tuck stitches was caused by its courses/wales ratio. However, the configurations in this study were selected in order to prevent consecutive tuck stitches, which are more challenging to model, as they resemble miss stitches.

### 4.2. Effect of Miss Stitches on a Weft Knit Sensor

**Figure 13:**
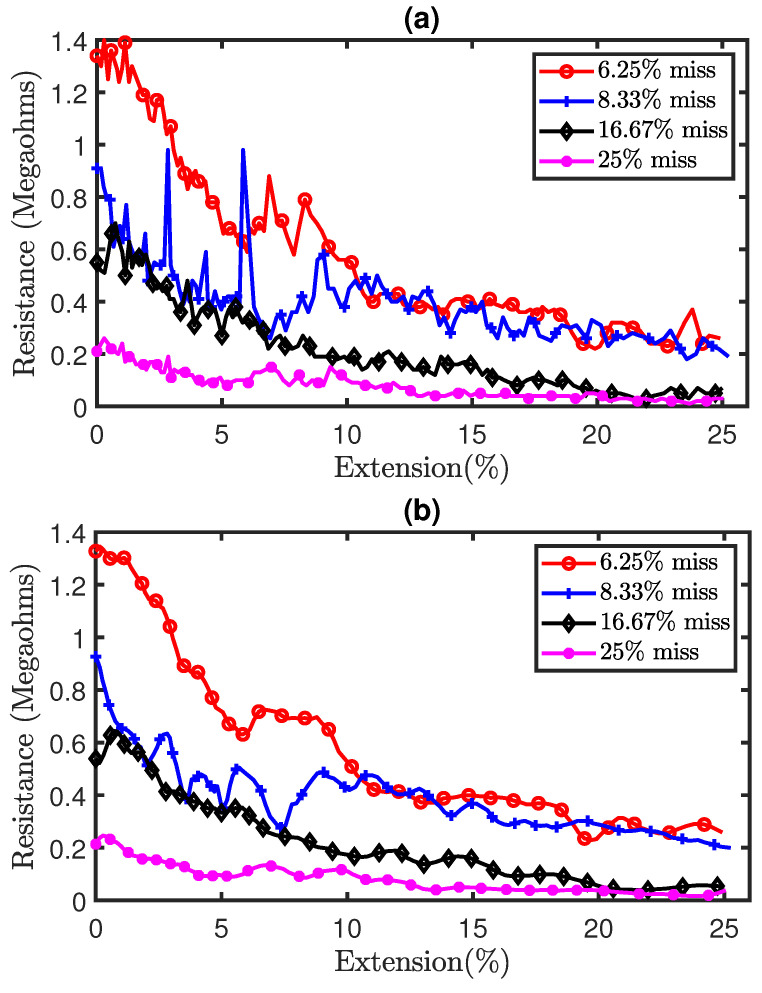
Experimental results of tensile test on sensors with miss stitches. (**a**) Pre-filtered results, (**b**) Post-filtered results.

**Figure 14:**
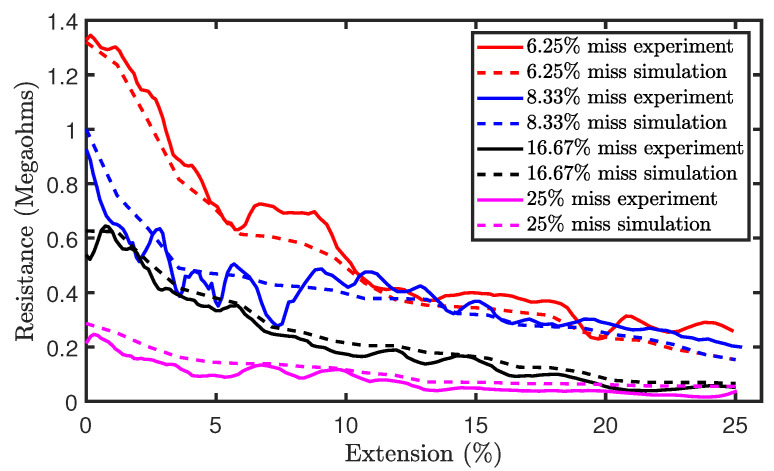
Comparison of simulation and experimental results for sensors with miss stitches.

Part (a) of Figure 13 shows the experimental results of the tensile test on sensors with miss stitches. The results illustrate the piezoresistivity of the sensors as their resistances decrease during extension. Moreover, the results also show significant noise as a result of hysteresis. Therefore, the Savitzky–Golay filter of polynomial order N = 5 and window length of 9 was also applied on the data in order to remove analog noise and the filtered results are shown in part (b) of Figure 13. In addition, Figure 14a shows a comparison of the simulation and experimental results. It can be observed that the simulation results generally agree with the experimental results thereby highlighting the accuracy of our proposed electromechanical models.

**Figure 15:**
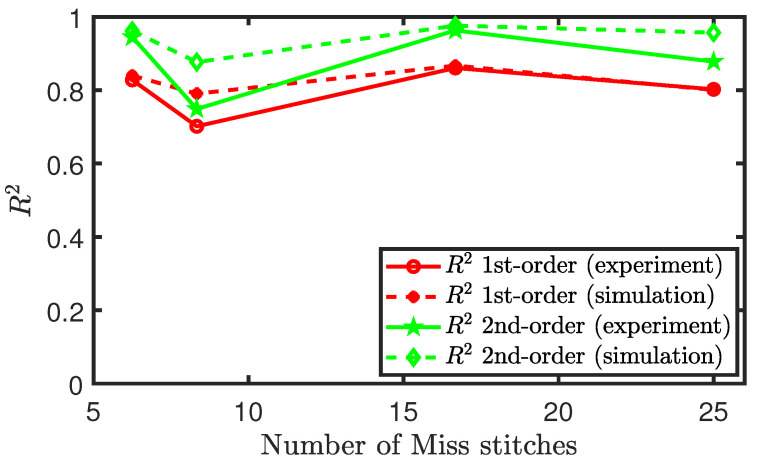
Polynomial fit of the piezoresistive behaviour of sensors with miss stitches.

Similar to the methodology that was used in analysing the experimental results of sensors consisting of tuck stitches, we plot the $R^{2}$ values, coefficient of determination, of both polynomial fits for each configuration of miss stitches in the sensor. We observe in Figure 15 that these plots exhibit a similar shape, but the polynomial fit of the second-order is better than the fit of the first order in terms of the $R^{2}$ value for every loop configuration consisting of miss stitches. However, we were unable to draw any consistent relationship between the changes in the miss stitches and the changes in the $R^{2}$ values of the sensors. This erratic behaviour is not well-modelled by our simulation results, because the presence of miss stitches causes the behaviour to be largely dependent on the floating stitch of the conductive yarn and not the weft knit structure of the sensor.

**Figure 16:**
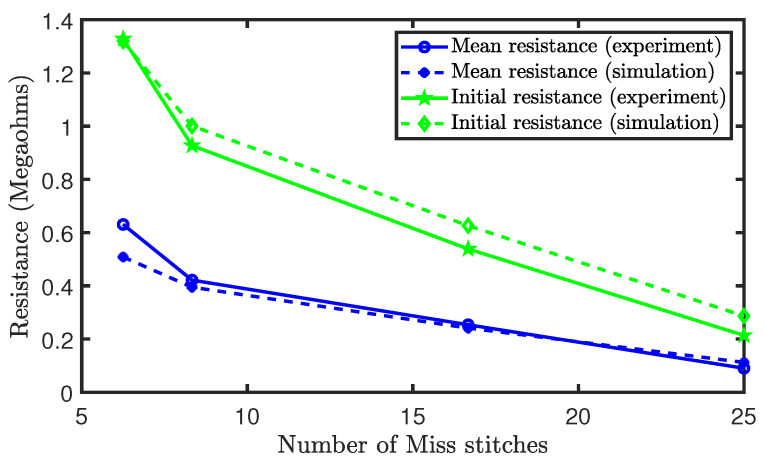
Initial and mean resistances of sensors with miss stitches.

Figure 16 depict the initial and mean resistance of the sensors during the experiment. It was shown that the initial resistance and the mean resistance reduces as the percentage of miss stitches increased. This occurred, because miss stitches do not interlock with other loops, leading to a lack of contact resistances at the locations of the missed stitches. Therefore, the increase in miss stitches reduces the contact resistances that are present in the sensor and, thus, the equivalent resistance of the sensor. Moreover, in terms of the initial and mean resistances, our simulation results closely agree with the experimental results.

## 5. Conclusions

In this paper, we have proposed two electromechanical models depicting a miss stitch and a tuck stitch in weft knit sensors. Subsequently, we expanded these models in order to simulate various loop configurations consisting of varying percentages of tuck or miss stitches in a weft knit sensor. The simulated results were then validated by tensile testing sensors that were knitted with the same simulated loop configurations and numerical parameters. It was observed that increases in the percentage of miss stitches or tuck stitches in a weft knit sensor decreased the initial resistance, mean, and median resistances. However, the sensor consisting of 16.67% tuck stitches did not agree with the simulated result in terms of the decrease of the mean resistance. It is believed that this occurred as a result of the limitations of the selected loop configuration.

Additionally, we observed that a quadratic polynomial best characterises the piezo-resistive behaviour of a weft knit sensor consisting of miss or tuck stitches. However, it was observed that increases in the percentage of tuck stitches in the sensor increased the $R^{2}$ value of the linear fit of its piezoresistivity ($R^{2}$ value of 0.954 for sensor with 25% tuck stitches). This is usually seen as a strong determinant of a weft knit sensor’s accuracy. Moreover, it ensures that machine learning classifiers can easily classify data that were acquired from a weft knit strain sensor in a wearable application. In contrast, increases in the percentage of miss stitches did not lead to a consistent change in the $R^{2}$ value of the sensor’s piezoresistivity.

This paper will provide researchers with fundamental knowledge regarding how to effectively model weft knit sensors with tuck or miss stitches. Moreover, the observations from the simulated and experimental results will direct the design and application of tuck or miss stitches on a weft knit sensor. In particular, researchers can utilise miss stitches in order to reduce the resistance of their weft knit strain sensors, and use tuck stitches to reduce the resistance and also increase the linearity of their weft knit strain sensors. Future work will entail the implementation of weft knit strain sensors comprising of 25% tuck stitches in a data glove.

## Figures and Tables

**Table 1:** Loop configuration of sensors. “**X**” represents a knitted loop stitch, “ ” represents a miss stitch and “·” represents a tuck stitch.

% of Miss/Tuck Stitches	Loop Configuration (Miss)	Loop Configuration (Tuck)
6.25%	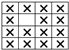	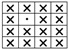
8.33%	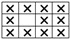	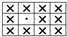
16.67%	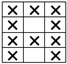	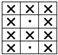
25%	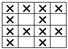	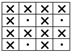

**Table 2:** Numerical Parameters for simulation.

Parameters	Values
Number of courses	72
Number of wales	72
$\alpha {(}^{\circ })$	24.75
$\beta {(}^{\circ })$	10.85
Course spacing (mm)	3
Wale spacing (mm)	2
ρ(ohms.mm)	300
Yarn’s Diameter (mm)	0.4

**Table 3:** Fabric parameters of the knitted sensors.

Configuration	Wales/cm	Courses/cm	Stitch Density
6.25% Miss	5.00	5.39	26.95
8.33% Miss	4.56	5.43	24.76
16.67% Miss	4.97	5.50	27.34
25% Miss	5.04	6.05	30.49
6.25% Tuck	4.11	5.48	22.52
8.33% Tuck	4.07	5.63	22.91
16.67% Tuck	3.60	6.49	23.64
25% Tuck	3.16	7.24	22.88

## References

[1] Xiang C., Guo J., Sun R., Hinitt A., Helps T., Taghavi M., Rossiter J. (2019). Electroactive textile actuators for breathability control and thermal regulation devices. Polymers.

[2] Almohammed B., Ismail A., Sali A. (2020). Electro-textile wearable antennas in wireless body area networks: Materials, antenna design, manufacturing techniques, and human body consideration—A review. Text. Res. J..

[3] Ferri J., Llinares Llopis R., Martinez G., Lidon Roger J.V., Garcia-Breijo E. (2020). Comparison of E-Textile Techniques and Materials for 3D Gesture Sensor with Boosted Electrode Design. Sensors.

[4] Atalay O., Kennon W.R., Demirok E. (2015). Weft-knitted strain sensor for monitoring respiratory rate and its electro-mechanical modeling. IEEE Sens. J..

[5] Patron D., Mongan W., Kurzweg T.P., Fontecchio A., Dion G., Anday E.K., Dandekar K.R. (2016). On the use of knitted antennas and inductively coupled RFID tags for wearable applications. IEEE Trans. Biomed. Circuits Syst..

[6] Isaia C., McMaster S.A., McNally D. Study of Performance of Knitted Conductive Sleeves as Wearable Textile Strain Sensors for Joint Motion Tracking. Proceedings of the 2020 42nd Annual International Conference of the IEEE Engineering in Medicine & Biology Society (EMBC).

[7] Wang J., Soltanian S., Servati P., Ko F., Weng M. (2020). A knitted wearable flexible sensor for monitoring breathing condition. J. Eng. Fibers Fabr..

[8] Li Y., Miao X., Raji R.K. (2019). Flexible knitted sensing device for identifying knee joint motion patterns. Smart Mater. Struct..

[9] Zhang H., Tao X., Wang S., Yu T. (2005). Electro-mechanical properties of knitted fabric made from conductive multi-filament yarn under unidirectional extension. Text. Res. J..

[10] Fan W., He Q., Meng K., Tan X., Zhou Z., Zhang G., Yang J., Wang Z.L. (2020). Machine-knitted washable sensor array textile for precise epidermal physiological signal monitoring. Sci. Adv..

[11] Ou J., Oran D., Haddad D.D., Paradiso J., Ishii H. (2019). SensorKnit: Architecting textile sensors with machine knitting. 3D Print. Addit. Manuf..

[12] Atalay O., Kennon W.R., Husain M.D. (2013). Textile-based weft knitted strain sensors: Effect of fabric parameters on sensor properties. Sensors.

[13] Atalay O., Kennon W. (2014). Knitted strain sensors: Impact of design parameters on sensing properties. Sensors.

[14] Holm R. (2013). Electric Contacts: Theory and Application.

[15] Atalay O., Tuncay A., Husain M.D., Kennon W.R. (2017). Comparative study of the weft-knitted strain sensors. J. Ind. Text..

[16] Spencer D.J. (2001). Knitting Technology: A Comprehensive Handbook and Practical Guide.

[17] McMaster S.A. (2018). Method for Making Electrically Conductive Textiles and Textile Sensor. U.S. Patent.

[18] Liu S., Liu Y., Li L. (2019). The impact of different proportions of knitting elements on the resistive properties of conductive fabrics. Text. Res. J..

[19] Liu S., Yang C., Zhao Y., Tao X.M., Tong J., Li L. (2016). The impact of float stitches on the resistance of conductive knitted structures. Text. Res. J..

[20] Postle R., Munden D. (1967). Analysis of the Dry-Relaxed Knitted-Loop Configuration: Part I: Two-Dimensional Analysis. J. Text. Inst..

[21] Kurbak A., Kayacan O. (2008). Basic studies for modeling complex weft knitted fabric structures part V: Geometrical modeling of tuck stitches. Text. Res. J..

[22] Munden D. (1959). The geometry and dimensional properties of plain-knit fabrics. J. Text. Inst. Trans..

[23] Zhang H., Tao X. From wearable to aware: Intrinsically conductive electrotextiles for human strain/stress sensing. Proceedings of the 2012 IEEE-EMBS International Conference on Biomedical and Health Informatics (BHI).

[24] Li L., Au W.M., Wan K.M., Wan S.H., Chung W.Y., Wong K.S. (2010). A resistive network model for conductive knitting stitches. Text. Res. J..

